# Editorial: Nutrigenomics: omics of maternal nutrition and foetal programming

**DOI:** 10.3389/fgene.2024.1327341

**Published:** 2024-03-08

**Authors:** Einar Vargas-Bello-Pérez, Ahmed Elolimy, Alzahraa M. Abdelatty

**Affiliations:** ^1^ Department of Animal Sciences, School of Agriculture, Policy and Development, University of Reading, Reading, United Kingdom; ^2^ Facultad de Zootecnia y Ecología, Universidad Autónoma de Chihuahua, Chihuahua, Mexico; ^3^ Animal Production Department, National Research Centre, Giza, Egypt; ^4^ Department of Nutrition and Clinical Nutrition, Faculty of Veterinary Medicine, Cairo University Giza, Giza, Egypt

**Keywords:** nutrigenomics, maternal nutrition, foetal programming, omics, animal and human models

The impact of nutrition on maternal and fetal health is enormous and can limit a newborn’s productive performance as well as metabolic functions such as those related to lipids and carbohydrates. Maternal nutrition has been taken to the molecular level, where the interplay between maternal functional nutrient uptake and fetal-Omics has brought to us several key pathways to better understand maternal nutrigenomics (effect of nutrition on gene expression at different tissue levels), fetal early-life programming (how maternal nutrition affects fetus development and newborns health), and maternal nutrition-fetal programming either through placenta or milk.

Nutrients have a deep impact on maternal-fetal programming and in-depth study of such mechanisms unravels the complexity of maternal-fetal nutrient-gene interactions and provides insight into fetal programming. The understanding of such mechanisms can prevent negative fetal health conditions during gestation or after birth. Therefore, the objective of this Research Topic entitled “*Nutrigenomics: Omics of Maternal Nutrition and Fetal Programming*” was to provide recent updates on nutrigenomics with a particular focus on their role in maternal health, fetal early-life programming, and the interplay between maternal nutrition and fetal programming. This Research Topic accepted four articles (3 research articles and 1 literature review) covering the aforementioned aspects.

It is known that maternal malnutrition negatively affects the offspring’s health due to epigenome changes. Current knowledge shows that the changes in DNA methylation could be modulated by supplementing a newborn’s diet. Therefore, Ando et al. supplied the diets of hypertensive pups with different quantities of protein after a state of fetal protein shortage to elucidate whether postnatal protein supplementation could change negative changes in DNA methylation in underfed gestating dams. They fed rat dams (during pregnancy) and their male offspring with diets with different protein inclusions. They reported that different dietary protein levels led to the reprogramming of different methylated DNA regions, and some other genes were readjusted when male offspring were fed with different protein amounts. Overall, the study revealed that postnatal nutrition (dietary protein) can modulate the epigenome.

It has been described that maternal nutrition during gestation affects gene expression in offspring. Thus, Sosa-Larios et al. evaluated the effect of limiting protein in maternal diets during gestation and postnatally analyzed the pancreatic islets of male progeny, from juveniles and young adults, in rats. Specifically, they analyzed the expression of genes involved in β-cell function and DNA methylation. In general, their study showed that lack of dietary protein during gestation alters genes related to β-cell function in male juvenile offspring while decreasing DNA methylation. Interestingly, this process could promote the improvement of β-cell function and affect the offspring’s health.

This Research Topic also published a review article from Harmancıoğlu and Kabaran that discussed the effects of high levels of maternal dietary fat on fetal epigenetic hypothalamic that results in obese newborns. From what was discussed in the review article, it seems that maternal nutrition is pivotal for improving the fetal metabolic environment with positive effects on the newborn’s health.

Lastly, an article from Argentato et al. was published in this Research Topic. They determined the relationship between genes related to methylation, offspring growth, and body composition. They reported different associations of genes related to methylation. For example, those relations were found in cord blood with centiles of fetal biparietal diameter and abdominal subcutaneous fat thickness, and newborn head circumference. This study showed how the epigenome plays an important biological role in the offspring’s growth and body composition.

In summary, the results from the above-mentioned studies have improved our understanding of maternal nutrition and its relation to fetal programming. In this Research Topic, dietary protein was the main cause of changes at the pancreatic level as well as offspring growth and body composition. It is important to note that all received articles used rodents as research models. Further studies should include other types of animals in different types of productive systems (i.e., cows, chickens, and sheep) as well as companion animals. This will provide wider insights into how maternal nutrition affects fetuses and newborns’ lives. Despite all existing scientific reports related to this Research Topic, greater efforts are still needed to prevent neonatal health and metabolic problems when there is maternal malnutrition. Overall, this research line is very important as the use of animal models can also help to prevent similar problems in humans (i.e., prevention of diabetes and obesity).

